# Sensitivity Improvement of Urchin-Like ZnO Nanostructures Using Two-Dimensional Electron Gas in MgZnO/ZnO

**DOI:** 10.3390/s19235195

**Published:** 2019-11-27

**Authors:** So-Young Bak, Jeongseok Lee, Yoojong Kim, Se-Hyeong Lee, Kyoungwan Woo, Sanghyun Lee, Moonsuk Yi

**Affiliations:** 1 Department of Electronics Engineering, Pusan National University, Busan 46241, Korealeejs6015@naver.com (J.L.); yoojongkim@pusan.ac.kr (Y.K.); shlee12@pusan.ac.kr (S.-H.L.); kwwoo200@pusan.ac.kr (K.W.); 2 Department of Smart Hybrid Engineering, Pusan National University, Busan 46241, Korea; collie20@pusan.ac.kr

**Keywords:** gas sensors, 2DEG, ZnO, MgZnO, vapor phase growth, sol-gel

## Abstract

This paper introduces a strategy for improving the sensitivity of a gas sensor to NO_2_ gas. The gas sensor was fabricated using urchin-like ZnO nanostructures grown on MgO particles via vapor-phase growth and decorated with MgZnO nanoparticles via a sol-gel process. The urchin-like ZnO gas sensor decorated with MgZnO showed higher sensitivity to NO_2_ gas than a pristine urchin-like ZnO gas sensor. When ZnO and MgZnO form a heterojunction, a two-dimensional electron gas is generated. This improves the performance of the fabricated gas sensor. The growth morphology, atomic composition, and phase structure were confirmed through field-emission scanning electron microscopy, energy-dispersive X-ray spectroscopy, and X-ray diffraction, respectively.

## 1. Introduction

Metal oxide semiconductors (MOSs) are widely used as a gas sensor material because of their simple structure, easy fabrication, low cost, semi-permanent use, and high reactivity to gases. In a MOS, there are n-type MOSs (where electrons act as charge carriers) and p-type MOSs (where holes act as charge carriers). The majority of MOS-type gas sensors use n-type MOSs, because they are faster than p-type MOSs [1,2,3].

**Figure 1:**
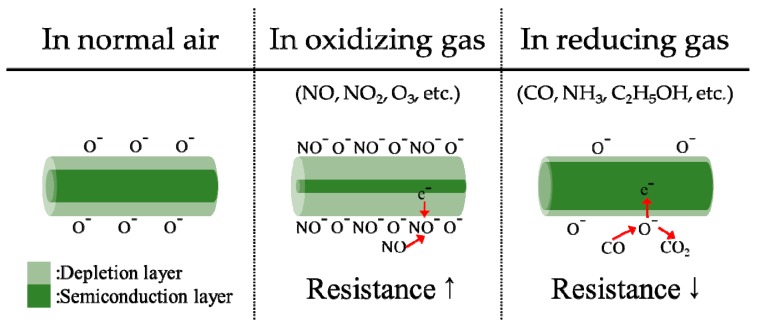
Schematic model of n-type metal oxide semiconductor (MOS) gas sensors in normal air, oxidizing gas, and reducing gas.

Oxygen vacancies are formed owing to nonstoichiometry in an n-type MOS. This causes oxygen ions (O^−^, O^2−^) to be attached on the surface of the MOS, thereby depriving electrons from 200 to 400 °C (Figure 1). The center of the MOS is still semiconducting, but the surface is deprived of electrons and forms an electrical depletion layer [4,5]. When exposed to oxidizing gases, the thickness of this depletion and resistance layer increase, because oxidizing gases take electrons from the MOS. In contrast, when exposed to a reducing gas, the resistance decreases, because the reducing gas returns electrons to the MOS during the course of the reaction with the adsorbed oxygen [6,7,8,9].

ZnO is a representative n-type wide-bandgap MOS material. It is highly efficient for gas sensing owing to its simple fabrication method, high sensitivity to toxic gases, and good biocompatibility [10,11]. When Zn is substituted with Mg in ZnO, MgZnO is synthesized, and the bandgap widens as the ratio of Mg increases [12]. Owing to the difference in radius between Zn and Mg, the lattice constant also changes. The ZnO crystal structure, which is a hexagonal wurtzite, is retained when x is less than 0.3 in Mg_x_Zn_1−x_O [13].

There are three key requirements for gas sensors, namely, selectivity, sensitivity, and speed. To improve these parameters, various methods, such as growing a hierarchical nanostructure, forming a heterojunction with p-type metal oxide semiconductors [14], and decorating with noble metal catalysts [15], have been studied. Vapor-phase growth is one of the ways to enhance performance, as it produces nanostructures with high surface-to-volume ratios. This method is suitable for gas sensors, because it allows for simple fabrication of various MOS-based nanostructures [16].

In this study, urchin-like ZnO nanostructures were grown on indium tin oxide (ITO) glass via vapor-phase growth and decorated with Mg_x_Zn_1−x_O (where x ranges from 0 to 0.3) nanoparticles. These structures were used as a gas sensor. The sensitivity of the gas sensor increased upon using a two-dimensional electron gas (2DEG) generated from a heterojunction of ZnO and MgZnO [17,18].

## 2. Materials and Methods

**Figure 2:**
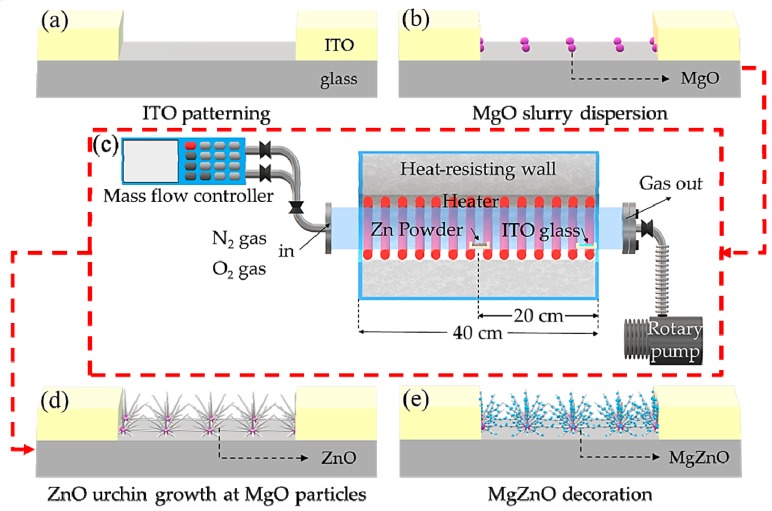
Fabrication steps: (**a**) patterning ITO substrates, (**b**) dropping MgO slurry, spin-coating, and drying to disperse MgO particles, (**c**,**d**) growing ZnO nanostructures, and (**e**) decorating with MgZnO.

The ITO glass substrate was cut to a size of 2 × 2 cm^2^. The interdigitated ITO electrodes on the glass substrate were patterned using photo lithography (Figure 2a).

To produce 0.025 mol/L MgO slurry, MgO powder (99.99%, Sigma-Aldrich Korea) and deionized (DI) water were poured into a vial and dispersed through ultrasonic treatment for 60 min. The substrate was immersed in buffered oxide etchant (BOE) for 3 s, rinsed with DI water, blown with N_2_ gas, and heated at 110 °C for 3 min to activate the surface. MgO slurry was dropped on the substrate, which was spin-coated at 2000 rpm for 30 s (Figure 2b).

As shown in Figure 2c, the alumina boat filled with 0.3 g of Zn powder (>150 μm, 99.995%, Sigma-Aldrich Korea) was located in the middle of the tube furnace. The substrate was placed in an alumina boat and positioned between 18 and 20 cm away from the center of the tube furnace. The temperature was raised from room temperature to 900 °C for 40 min while N_2_ gas (N_2_: 100 sccm) was flown. The ZnO nanostructures were grown for 1 h via a reaction between the source powder and an N_2_-O_2_ mixed gas (N_2_: 100 sccm, O_2_: 0.2 sccm), and subsequently, they were slowly cooled. Through this process, urchin-like ZnO nanostructures grown at MgO were obtained (Figure 2d) [19].

The concentration of the mixture of Zn and Mg was fixed at 0.05 mol/L, and the concentration of Mg was changed from 0 to 0.015 mol/L. Zinc acetate dihydrate (Zn(CH_3_COO)_2_·2H_2_O, 99.999%, Sigma-Aldrich) and magnesium acetate tetrahydrate (Mg(CH_3_COO)_2_·4H_2_O, 99%, Sigma-Aldrich) were dissolved in 2-methoxyethanol as a solvent, and 0.2 mol/L of ethanolamine was added as a stabilizer. The solution was stirred at 70 °C and 400 rpm for 2 h and aged at room temperature for 24 h. Then, the solution was dropped on the ZnO nanostructures, and the sample was spin-coated at 3000 rpm for 30 s. The coated samples were dried at 100 °C on a hot plate and annealed at 600 °C in a tube furnace for 30 min (Figure 2e). The following chain of chemical reactions produced the formation of MgZnO nanoparticles via a sol-gel process [20]:

Mg(CH_3_COO)_2_ 4H_2_O + Zn(CH_3_COO)_2_ · 2H_2_O → Mg_x_Zn_1−x_(OH)_2_ + CH_3_COOH (1)

Mg_x_Zn_1−x_(OH)_2_ + Mg_x_Zn_1−x_(OH)_2_ → Mg_x_Zn_1−x_O + H_2_O (2)

Mg_x_Zn_1−x_(OH)_2_ + Mg_x_Zn_1−x_(CH_3_COO)_2_ → Mg_x_Zn_1−x_O + CH_3_COOH. (3)

For simplicity, the samples are denoted using the ratio of Mg to the simple integer ratio of Mg and Zn. For example, Mg_0.1_Zn_0.9_O-decorated urchin-like ZnO is referred to as 1M-ZnO, whereas Mg_0.2_Zn_0.8_O-decorated urchin-like ZnO is referred to as 2M-ZnO.

## 3. Results and Discussion

### 3.1. Material Analysis

#### 3.1.1. Field-Emission Scanning Electron Microscopy (FE-SEM) and Energy-Dispersive X-ray Spectroscopy (EDS) Analyses

**Figure 3:**
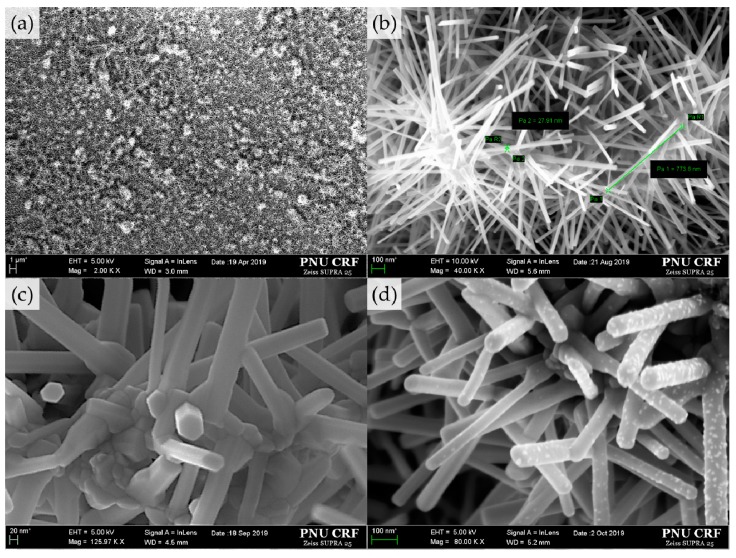
Field-emission scanning electron microscopy (FE-SEM) image of (**a**) urchin-like ZnO nanostructures growing evenly across the substrate, (**b**) urchin-like ZnO networks, (**c**) tips of nanowires, and (**d**) urchin-like ZnO decorated with MgZnO.

The morphologies of the specimens were analyzed using field-emission scanning electron microscopy (FE-SEM, SUPRA25, ZEISS, Germany). Figure 3a shows that the nanostructures were networked together. The urchin-like ZnO nanostructures consist of multiple nanowires that grow together from a central point. In Figure 3b, the nanowires were observed to have a thickness between 30 and 50 nm and a length between 600 and 800 nm. The tip of the nanowires is a hexagon without round liquid catalyst droplets. This indicates that the structures were grown via a vapor–solid process (Figure 3c). Figure 3d shows the nanostructures with the added 0.05 M MgZnO nanoparticles, which were shaped as the urchin in Figure 3c. The fact that the structure did not collapse indicates that the surface-to-volume ratio was almost maintained.

**Figure 4:**
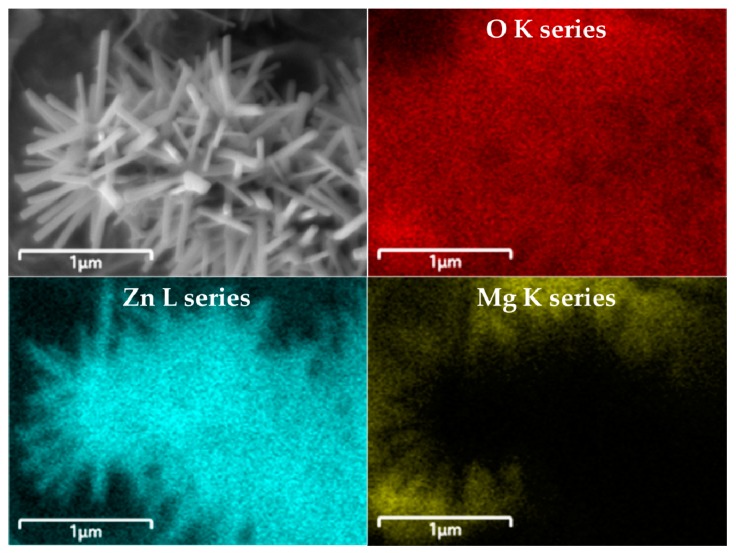
Energy-dispersive X-ray spectroscopy (EDS) mapping of urchin-like ZnO decorated with MgZnO.

The elements of the samples were analyzed using energy-dispersive X-ray spectroscopy (EDS, Ultim Max, Oxford Instruments, UK). The EDS mapping images are shown in Figure 4. It was observed that the urchin-like nanostructures were mainly composed of Zn and O. As Mg was employed as a nanoparticle for decoration, it was detected in the surroundings of the nanostructure. Similarly, O was found in the whole sample, because it was included in both structures and decoration materials.

#### 3.1.2. XRD Analysis

**Figure 5:**
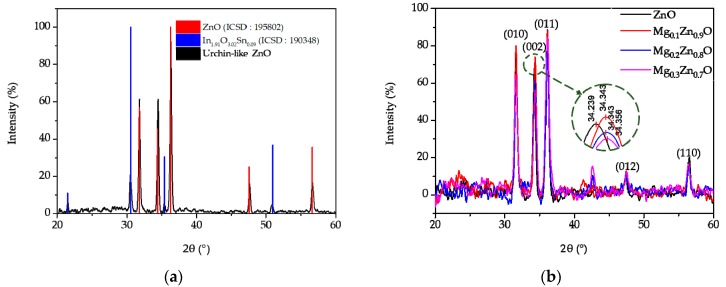
XRD analyses of (**a**) urchin-like ZnO and (**b**) ZnO, Mg_0.1_Zn_0.9_O, Mg_0.2_Zn_0.8_O, and Mg_0.3_Zn_0.7_O.

In Figure 5a, the crystal structures of the samples were analyzed using X-ray diffraction (XRD). The XRD pattern was indexed to hexagonal wurtzite ZnO (ICSD number 195802) and In_1.91_O_3.02_Sn_0.09_ (ICSD number 190348). This confirmed that ZnO existed on the gas sensor and that ITO remained after the vapor-phase growth. Even if the composition ratio varied from ZnO to Mg_0.3_Zn_0.7_O, the hexagonal wurtzite structure was maintained, but the lattice constant changed owing to the difference in the radius of the element. Therefore, depending on the amount of Mg substituting Zn, the peak of the XRD graph shifted to the right or left [21]. In particular, depending on the Mg content, the (002) peak shifted to the right from 34.239 to 34.356 (Figure 5b).

### 3.2. Gas-Sensing Mechanism

**Figure 6:**
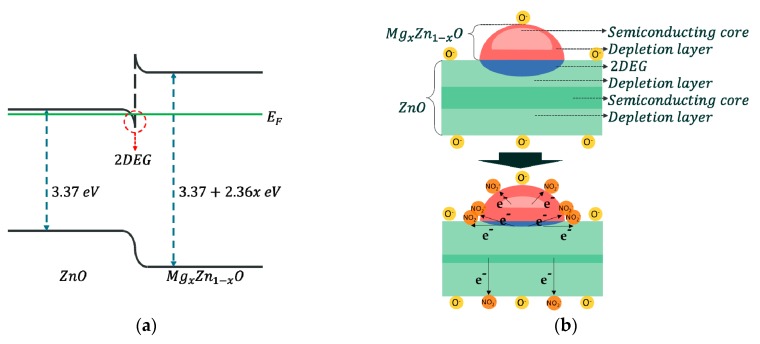
(**a**) Energy diagram of MgZnO/ZnO and (**b**) schematics of MgZnO/ZnO when exposed to an oxidizing gas.

The bandgap of MgZnO becomes wider as the Mg ratio increases [12]. When a heterojunction is formed by ZnO and MgZnO, which has a sufficiently wide bandgap, the electrons transfer from MgZnO to ZnO, and the Fermi level overpasses the conduction band at ZnO near the interface. As a result, a 2DEG is generated (Figure 6a). Electrons in the 2DEG region have very high mobility and quantized energy levels in one dimension but move freely in the remaining two dimensions [22]. Compared with pristine ZnO, a sudden change in resistance occurs when the sensor is exposed to oxidizing gases because the number of electrons that can participate in the reaction increases (Figure 6b). In other words, 2DEG improves the sensitivity and speed of gas sensors.

**Figure 7:**
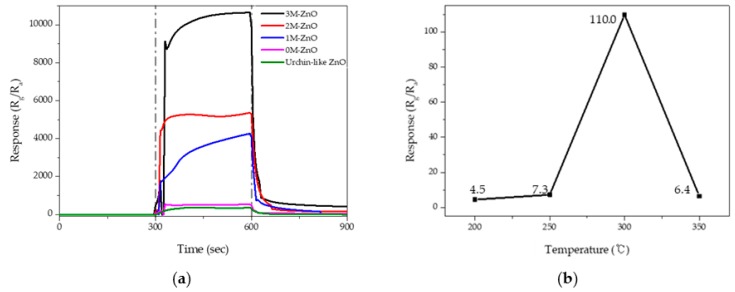
(**a**) Response of the urchin-like ZnO, 0M-ZnO, 1M-ZnO, 2M-ZnO, and 3M-ZnO to 100-ppm NO_2_ gas at 300 °C and (**b**) response of 3M-ZnO to 1-ppm NO_2_ according to temperatures from 200 to 350 °C.

The response of urchin-like ZnO nanostructures 0M-ZnO, 1M-ZnO, 2M-ZnO, and 3M-ZnO to 100-ppm NO_2_ gas was evaluated at 300 °C. The gas-sensing characteristics were investigated in a cleanroom where the relative humidity was kept below 50%. Before starting the reaction of the samples with NO_2_ gas, at 300 °C, N_2_ gas was injected until the samples were stabilized. This process is shown on the x-axis in Figure 7a from 0 to 300 s. The change of response was observed by exposing 100-ppm NO_2_ gas for 300 s. Then, the NO_2_ gas was removed at 600 s, and the resistance recovered its original state. The response of gas sensors was defined as R_g_/R_a_, where R_a_ and R_g_ are the resistances in N_2_ gas and in target gas, respectively.

The response of gas sensors to 100-ppm NO_2_ gas was measured at 300 °C (Figure 7a and Table 1). The gas response of the urchin-like ZnO nanostructures was relatively low, 367.9. In 0M-ZnO, this parameter was improved, reaching a value of 549.9, but the rate of increase was smaller than for the other counterparts. The response of the 1M-ZnO sensor to 100-ppm NO_2_ was 4264.3. The 2M-ZnO nanostructures provided an enhanced gas response of 5367.4, which was 14.6 times higher than the response of pristine ZnO nanostructures. The highest NO_2_ response was observed in the 3M-ZnO sensor. In this case, the response of the sample was 10,651.3, which was 29.0 times higher than that of the undecorated ZnO sample. When decorating MgZnO with a higher Mg ratio, the thickness of the 2DEG was increased, and the response was improved.

The response/recovery times were defined as the times to reach 90% of the final equilibrium value. As shown in the inset of Table 1, the response and recovery times of the urchin-like ZnO were 90 and 65 s, respectively. The response and recovery times of the 1M-ZnO, 2M-ZnO, and 3M-ZnO samples were shorter than that of the urchin-like ZnO.

To determine the optimum operating temperature, the response of the 3M-ZnO to 1-ppm NO_2_ was evaluated as a function of operating temperatures, as shown in Figure 7b. The response rapidly increased at 300 °C and then decreased with a further rise in the operating temperature.

The response of the samples to 100-ppm CO and NH_3_ at 300 °C are shown in Table 2. The response to CO and NH_3_ improved as the ratio of Mg increased but were insignificant compared with NO_2_. The response of MgZnO-decorated ZnO increased in the order of NO_2_ > NH_3_ > CO under the same conditions, showing good NO_2_ selectivity relative to CO and NH_3_.

**Figure 8:**
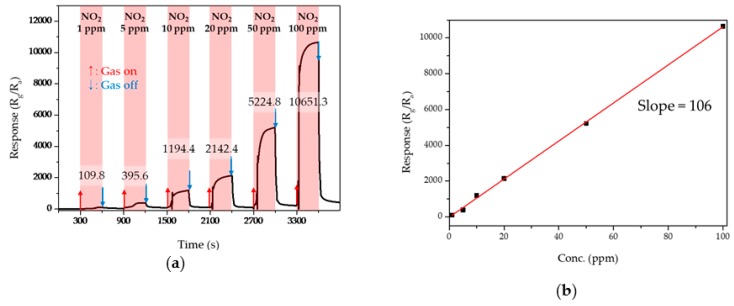
(**a**) Dynamic response curves of 3M-ZnO with different gas concentrations of NO_2_ and (**b**) linear fitted response as a function of NO_2_ concentration.

In Figure 8, the sensing transients of 3M-ZnO to 1–100-ppm NO_2_ were measured at 300 °C. The plot was linear, and the response to 1-ppm NO_2_ was high, with a value of 110. The relationship between the response to NO_2_ and the concentration of NO_2_ was nearly linear (with a coefficient of determination R^2^ = 0.99), as shown Figure 8b. The slope of the concentration-to-response curve was calculated to be 106 using a linear least-squares fit. The detection limit of NO_2_ was calculated to be 0.24 ppm from the plot when R_g_/R_a_ > 1.5 was used as the criteria for reliable gas detection. A brief summary of some examples about current development of ZnO based gas sensors is listed in Table 3. The observed response of 3M-ZnO was found to be significantly enhanced compared with other ZnO-based gas sensors.

## 4. Conclusions

We fabricated urchin-like ZnO nanostructures decorated with MgZnO via vapor-phase growth and a sol-gel process. The gas-sensing characteristics were investigated, and the material was analyzed. When exposed to an oxidizing gas, not only electrons in the core portion of ZnO and MgZnO but also electrons in the 2DEG region participated in the reaction, thereby giving rise to a rapid and sensitive reaction. The higher the proportion of Mg, the higher the number of electrons that were induced, indicating higher reactivity. The response and recovery time were decreased by the decoration of MgZnO on ZnO. The 3M-ZnO sensor demonstrated a high response (R_g_/R_a_ = 10,651) to 100-ppm NO_2_ at 300 °C, a value which is 29 times higher than the response of undecorated urchin-like ZnO.

## Figures and Tables

**Table 1:** The response, response time, and recovery time of gas sensors to 100-ppm NO_2_ gas at 300 °C.

Sample	Response (R_g_/R_a_)	Response Time (s)	Recovery Time (s)
Urchin-like ZnO	367.9	90	65
0M-ZnO	549.9	95	70
1M-ZnO	4264.3	75	65
2M-ZnO	5367.4	45	60
3M-ZnO	10651.3	60	30

**Table 2:** Response of the urchin-like ZnO, 0M-ZnO, 1M-ZnO, 2M-ZnO, and 3M-ZnO to 100-ppm CO and NH_3_ gas at 300 °C.

Target Gas, Concentration	Urchin-like ZnO	0M-ZnO	1M-ZnO	2M-ZnO	3M-ZnO
CO, 100-ppm	1.1	1.5	1.5	1.6	2.2
NH_3_, 100-ppm	1.1	1.6	1.6	1.8	2.4

**Table 3:** A brief summary of the sensor response of ZnO-based gas sensors.

Material	Structure	Target Gas, Concentration (ppm)	Operating Temperature (°C)	Gas Response	Ref.
ZnO	nanoflower	C_2_H_5_OH, 400	350	30.4	[23]
Ag-embedded ZnO	nanorod	C_2_H_5_OH, 50	280	34.8	[15,24]
Co-doped ZnO	nanorod	NO_2_, 500	210	88	[25]
Cr-doped ZnO	nanorod	C_2_H_5_OH, 400	300	45	[26]
Mg-doped ZnO	urchin	C_2_H_5_OH, 5	350	343.0	[19]
NiO-decorated ZnO	nanowire	HCHO, 5	450	10.03	[27]
α-Fe_2_O_3_-decorated ZnO	nanowire	CO, 100	300	18.8	[28]
MgZnO-decorated ZnO	Urchin	NO_2_, 100	300	10651.3	This work
